# Leptin and adiponectin dynamics at patients with rectal neoplasm - Gender differences

**DOI:** 10.1371/journal.pone.0212471

**Published:** 2019-08-19

**Authors:** Alexandru Florescu, Dumitru Branisteanu, Stefana Bilha, Dragos Scripcariu, Ioana Florescu, Viorel Scripcariu, Gabriel Dimofte, Ioana Grigoras

**Affiliations:** 1 Department of Endocrinology, “Grigore T. Popa” University of Medicine and Pharmacy, Iasi, Romania; 2 Department of Surgery, “Grigore T. Popa” University of Medicine and Pharmacy, Iasi, Romania; 3 Department of Intensive Care, “Grigore T. Popa” University of Medicine and Pharmacy, Iasi, Romania; Academy of Sciences of the Czech Republic, CZECH REPUBLIC

## Abstract

**Background:**

Numerous studies associate adipokines with colorectal malignancy, but few data deal with patients suffering exclusively of rectal carcinoma (RC).

**Aims:**

We evaluated leptin and adiponectin levels in RC patients compared to healthy population and their dynamics after surgery.

**Material and methods:**

Serum leptin and adiponectin were evaluated before surgery in 59 RC consecutive patients (38 males and 21 females), and in age and weight matched healthy controls. Measurements were repeated at 24, 72 hours and 7 days after surgery.

**Results:**

Adipokine levels were higher in women. Controls had higher leptin (32.±4.34 vs 9.51±1.73 ng/ml in women and 11±2.66 vs 2.54±0.39 ng/ml in men, p = 0.00048 and 0.0032) and lower adiponectin (9±0.64 vs 11.85±1.02 μg/ml in women and 7.39±0.51 vs 8.5±0.62 μg/ml in men, p = 0.017 and 0.019) than RC patients. Surgery caused an increase of leptin from 5.11±0.8 to 18.7±2.42 ng/ml, p = 6.85 x 10¨^8^, and a decrease of adiponectin from 9.71±0.58 to 7.87±0.47 μg/ml, p = 1.4 x 10¨^10^ for all RC patients and returned thereafter to the initial range at 7 days. Adipokines were correlated with body weight (BW). The significance of correlation persisted after surgery only in males, but disappeared in females. Adipokines were not modified by tumor position, presurgical chemoradiotherapy or surgical technique. Women with RC experiencing weight loss had higher adiponectin than women without weight modifications (p<0.05 at all time points).

**Conclusions:**

Adipokine levels of patients with RC differ from the healthy population, possibly reflecting an adaptation to disease. Adipokine modifications after surgery may be related to acute surgical stress. Whether leptin and adiponectin directly interact is not clear. Women have higher adipokine levels, more so after significant weight loss, but the strength of their correlation with BW decreases after surgery. These data suggest gender differences in the adipokine profile of RC patients which may find clinical applications.

## Introduction

Adipose tissue performs an endocrine role, mediated by hormones (adipokines) which are specifically secreted by the adipocytes [1]. Epidemiological data consistently show an association between weight gain and the risk of colorectal cancer in adults. This observation led to the evaluation of a possible linking role of adipokines with cancer pathogenesis [2–4]. Leptin is an adipokine which reflects fat mass, being involved in nutrition behavior, and is considered a proinflammatory and carcinogenic factor in various cancer types, stimulating carcinogenic angiogenesis [2,4–6]. Adiponectin is inversely related to fat mass and stimulates insulin secretion [1,2] and has an anti-inflammatory role, inhibiting angiogenesis and being considered protective against various cancer types [2,4,5]. Therefore adipokines are interesting candidates as biomarkers in colorectal cancer (CRC) with potential significance in cancer development and prognostic evaluation.

Literature shows conflicting data regarding leptin and adiponectin levels in digestive cancers. Different authors described increased [2,3,6–8], decreased [9–13] or unchanged leptin levels [4,14] in patients with CRC whereas adiponectin was found either decreased [2–4,8,15,16] or unmodified [17,18]. These discrepancies may be due to the fact that carcinogenesis is not only related to serum adipokine concentrations but also to adipokine receptors expressed on various cells, i.e. tissue sensitivity [11,18,19]. It is therefore unclear whether modifications in adipokine levels described in patients with digestive cancer represent a causative link or are merely an adaptive epiphenomenon [20]. Finally, the contrasting data described in the literature may be caused by the heterogeneity of studied groups with respect to tumor localization [3,9, 21,22], gender [7,23], weight modification [3,9], disease severity [8], chemotherapy [24] or due to statistical complexity caused by multicentricity of the study or complex metaanalysis [3,4,18,21,22]. In order to avoid these variations, one needs to choose a more homogenous series, in a single location at similar stages. Such type of neoplasia is the carcinoma located in the rectal region (rectal carcinoma, RC) which is usually not accompanied by cachexia, a problem that might affect changes in adipokines.

The aims of our study were therefore to observe differences in leptin and adiponectin levels between patients suffering of RC operated in the same surgical center and healthy age, weight and gender-matched controls. We also intended to follow the evolution of leptin and adiponectin after surgery and at distance, at different time points.

## Materials and methods

### Study participants

The study was approved by the Ethical Committee of Clinical Research of the Regional institute of Oncology of Iasi (nr. 167 from 30/06/2017). All patients and controls were informed about the study procedures and signed a written informed consent.

**Fig 1:**
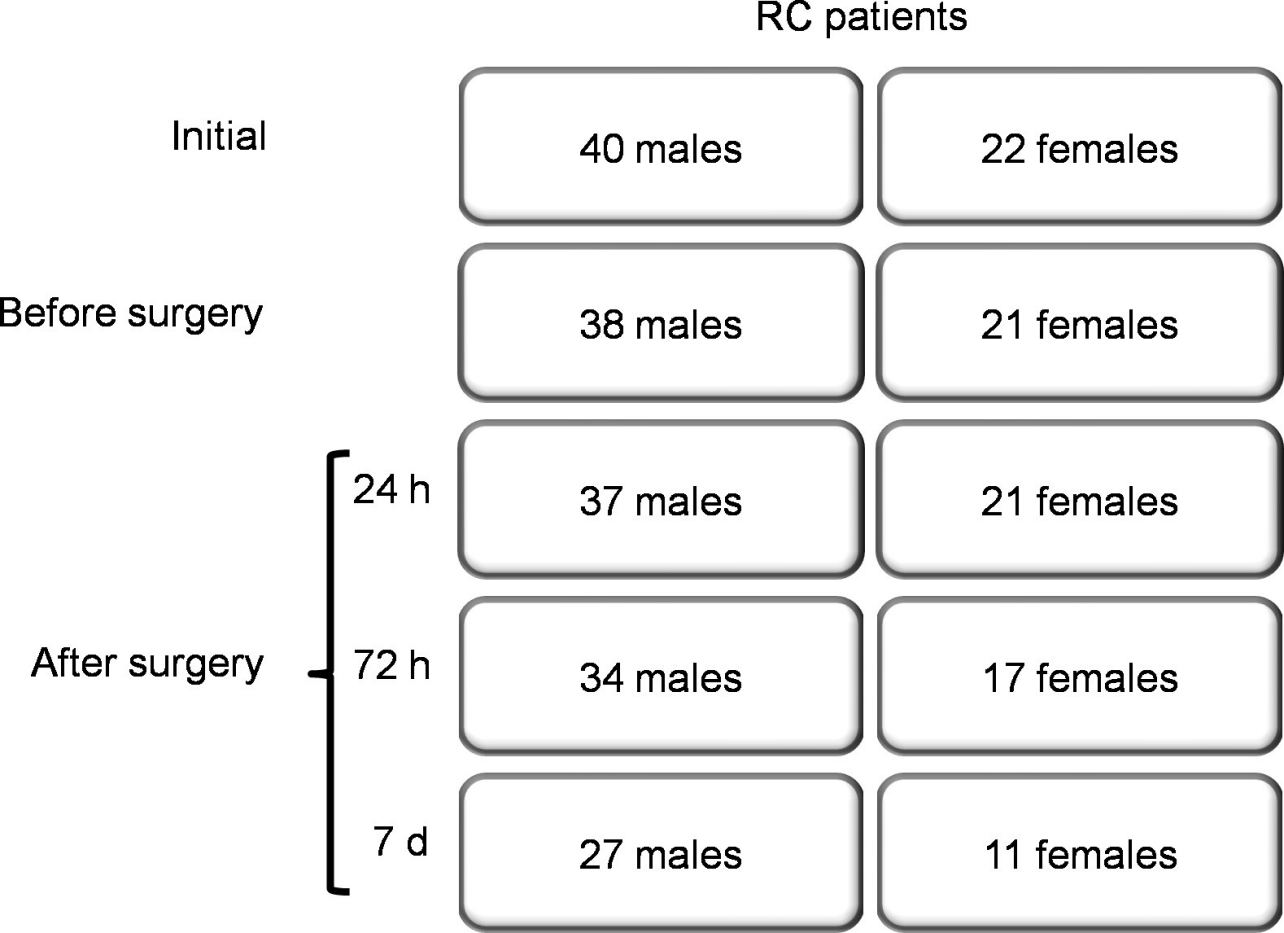
Recruitment and dropout diagram. RC = rectal carcinoma.

We recruited between August 2017 and August 2018 an initial number of 62 patients (40 males and 22 females) diagnosed with stage 2 and 3 RC and consecutively programmed for surgical intervention at the Regional Institute of Oncology of Iasi. Exclusion criteria were stage 4 invasive RC with distant metastases, extreme obesity (BMI > 35 kg/m^2^), and diabetes mellitus. Thirty patients followed neoadjuvant chemoradiotherapy (nCRT) for 2 months before surgery (pelvic irradiation of 45 Gy and concomitant Capecitabine 1600–1650 mg/m^2^/day) [25] and were operated 60 ± 10 days after chemoradiotherapy arrest, but two males and one female of chemoradiotreated patients dropped out from the study before surgery. The other 32 patients were operated directly, without nCRT. Patients were submitted to various techniques of surgical intervention, according to location within the rectum: low anterior resection type operations (LAR) in 42 patients and abdomino-perineal resection type (APR) in other 17 patients. Blood samples were collected before surgery, as well as at 24 hours, 72 hours and 7 days after surgery, in order to evaluate serum leptin and adiponectin levels. Other 20 patients (10 males and 10 females) gradually dropped out during the follow up period, due to refusal to further participate, withdrawal from the informed consent or absence at routine check-up 7 days after surgery (Fig 1).

Serum leptin and adiponectin were also evaluated in 29 male and 28 female age and weight-matched healthy controls, with no malignant antecedents. All patients and controls were weighed with the same weighing scale. Patients with RC were questioned about their body weight (BW) evolution during the year before enrollment. The characteristics of RC patients and of controls are depicted in Table 1.

**Table 1:** Characteristics of RC patients and controls.

	Males			Females		
	RC	Control	p	RC	Control	p
Number	38	29	-	21	28	-
Age (years, mean ± SD)	66.1 ± 8.3	63.9 ± 6.8	0.203	67.9 ± 8.4	66.8 ± 5.5	0.469
Weight (kg, mean ± SD)	77.9 ± 11.3	81.5 ± 19.7	0.273	72.2 ± 11.4	76.5 ± 14.7	0.221
BMI (kg/m^2^, mean ± SD)	25.1 ± 3.2	26 ± 3.6	0.301	26.5 ± 3.8	27.4 ± 4.1	0.263

### Evaluation of serum leptin and adiponectin

After overnight fasting, a sample of 10 ml blood was drawn from forearm vein from all study participants and the separated serum aliquots were stored at -20°C until assessment. Blood collection of RC patients was performed before surgery and repeated 24 hours, 72 hours and seven days after surgery. Serum assessment of human leptin and adiponectin was performed by using magnetic high performance technology (Luminex Screening Assay, R&D Systems Inc., MN, USA) with a sensitivity limit of 10 pg/mL for leptin and of 148 pg/mL for adiponectin.

### Statistical analysis

Data are expressed as mean ± standard error of the mean (SEM). SPSS (statistics version 20.0 for Windows) was used for statistical analysis. Shapiro-Wilk test was used to verify the normal distribution of data. Differences between groups were tested using the student’s t test and the Mann-Whitney U test. Correlation analysis (Pearson analysis for normally distributed data and Spearman rank correlation for skewed data) was performed to investigate the relationship between leptin and adiponectin, as well as between BW and leptin or adiponectin, respectively. The possibility of BW of being a confounder in the relationship between leptin and adiponectin was investigated through hierarchical regression. Differences were considered significant at p values < 0.05.

## Results

### Comparison of mean leptin and adiponectin levels between RC patients and healthy controls

Females had higher leptin and adiponectin levels than males in both control and RC groups. When compared to controls, RC patients had significantly lower leptin and higher adiponectin levels in both sexes (Table 2).

**Table 2:** Differences in adipokine values between patients with RC before surgery and healthy controls and between males and females.

Parameter	CTR males	RC males	p values CTR vs RC males	CTR females	RC females	p values CTR vs RC females
Leptin (ng/ml)	11 ± 2.6 ***	2.5 ± 0.4**	*p = 0*.*0032*	32.2 ± 4.3	9.5 ± 1.7	*p = 0*.*00049*
Adiponectin (μg/ml)	7.4 ± 0.5*	8.5 ± 0.6**	*p = 0*.*019*	9± 0.6	11.9 ± 1	*p = 0*.*0171*

CTR–healthy control groups; RC–patients with rectal carcinoma.

* p < 0.05

** p<0.01

***p<0.001 compared to females.

### Dynamics of leptin and adiponectin after surgical intervention for RC

**Fig 2:**
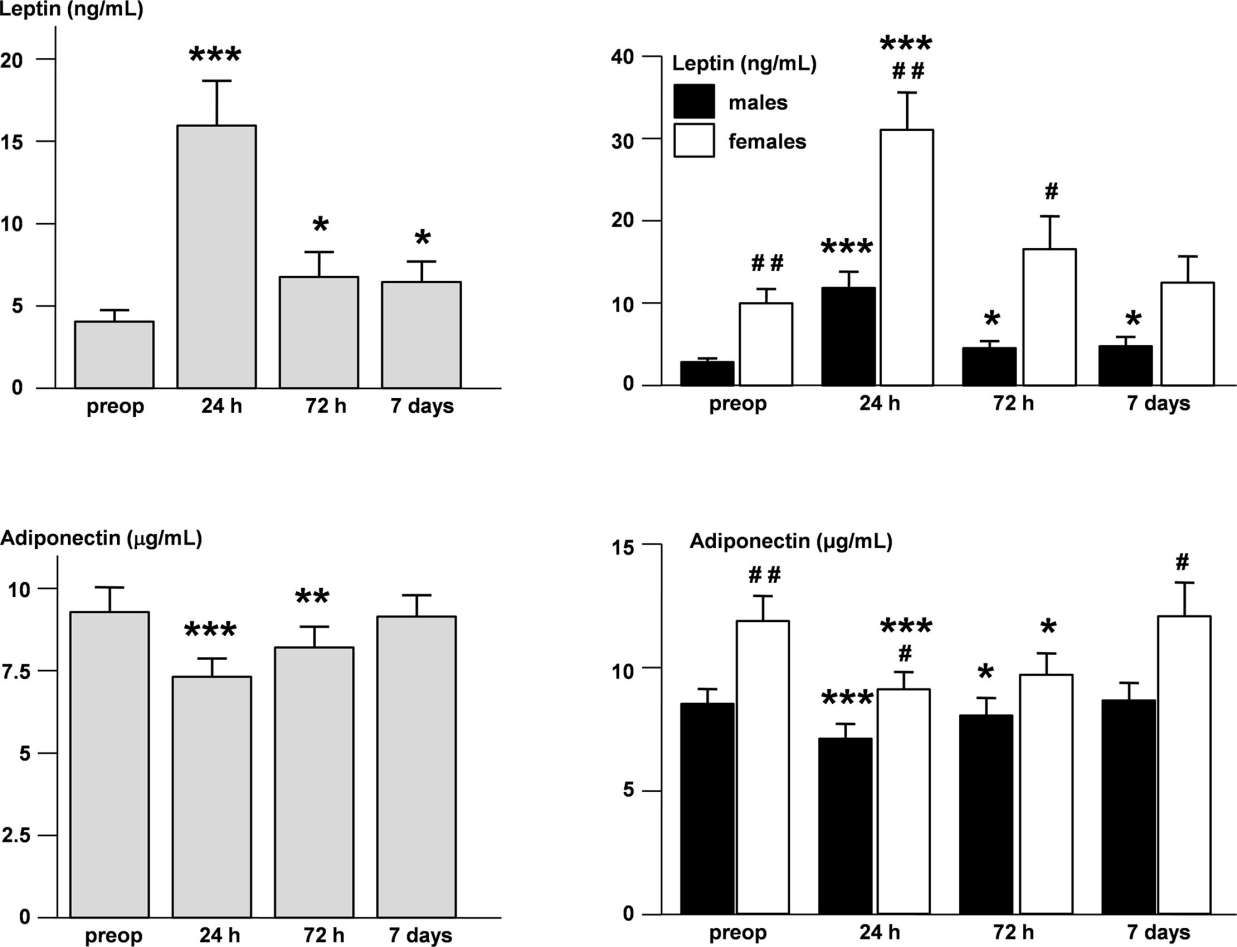
Dynamics of leptin and adiponectin after surgery. Leptin (upper panel) and adiponectin (lower panel) levels (mean +/- SEM) in all patients with RC (left, grey bars) and in males (right, black bars) or females only (right, white bars) before surgery and at 24, 72 hours and 7 days after surgery. * compared to presurgical values. # compared to males, *** p < 0.0001, ** or ## p < 0.01, * or # p < 0.05.

Leptin and adiponectin of RC patients showed a mirror profile after surgery. They displayed a steep increase of leptin (from 5.11 ± 0.8 to 18.7 ± 2.42 ng/ml, p = 6.85 x 10^−8^) and a significant decrease of adiponectin (from 9.71 ± 0.58 to 7.87 ± 0.47 μg/ml, p = 1.4 x 10^−10^) 24 hours after surgery. The levels of the two adipokines recovered to presurgical levels during the follow up period. The dynamics of the two adipokines is preserved when data are analyzed separately in the male and female subgroups (Fig 2).

### Correlations between leptin, adiponectin and BW

BW did not change significantly during the follow-up week. Adipokines were correlated to BW before surgery in both males and females. Leptin was positively correlated, whereas adiponectin was inversely correlated. Surgery caused a persistent loss of correlation significance of leptin and adiponectin with BW in females, but not in males (Table 3).

**Table 3:** Correlation coefficients of BW with leptin and adiponectin in males and females with RC.

Gender	time	nr	Leptin		Adiponectin	
			r^2^	p	r^2^	p
Males	Before surgery	38	0.13	***0*.*027***	0.11	***0*.*045***
	24 h	37	0.132	***0*.*031***	0.159	***0*.*016***
	72 h	34	0.139	***0*.*028***	0.116	***0*.*048***
	7 days	27	0.322	***0*.*00056***	0.144	***0*.*049***
Females	Before surgery	21	0.263	***0*.*021***	0.239	***0*.*025***
	24 h	21	0.045	0.359	0.132	0.106
	72 h	17	0.088	0.247	0.15	0.125
	7 days	11	0.041	0.549	0.135	0.267

r^2^ = regression coefficient of determination. With italic bold–significant correlations.

**Fig 3:**
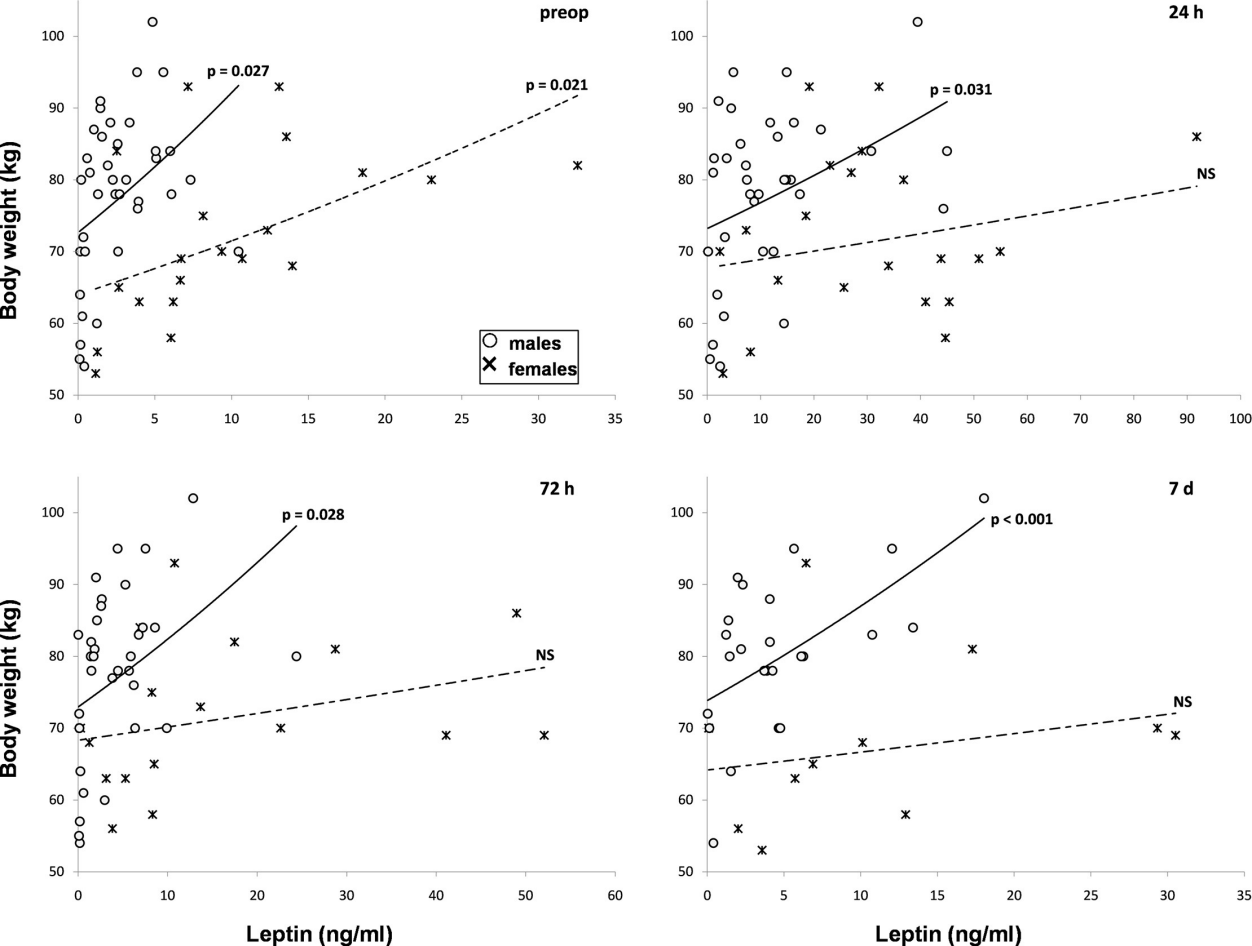
Correlations between weight and serum leptin in males (○) and females (×). Correlations before surgery (preop, up, left), after 24 hours (up, right), 72 hours (low, left) and seven days (low, right). Correlation was considered significant at p values lower than 0.05. NS = non-significant.

The gender difference of correlation slopes was obvious for the leptin-BW correlation at all post-surgical time points (Fig 3).

### Correlations between leptin and adiponectin

**Fig 4:**
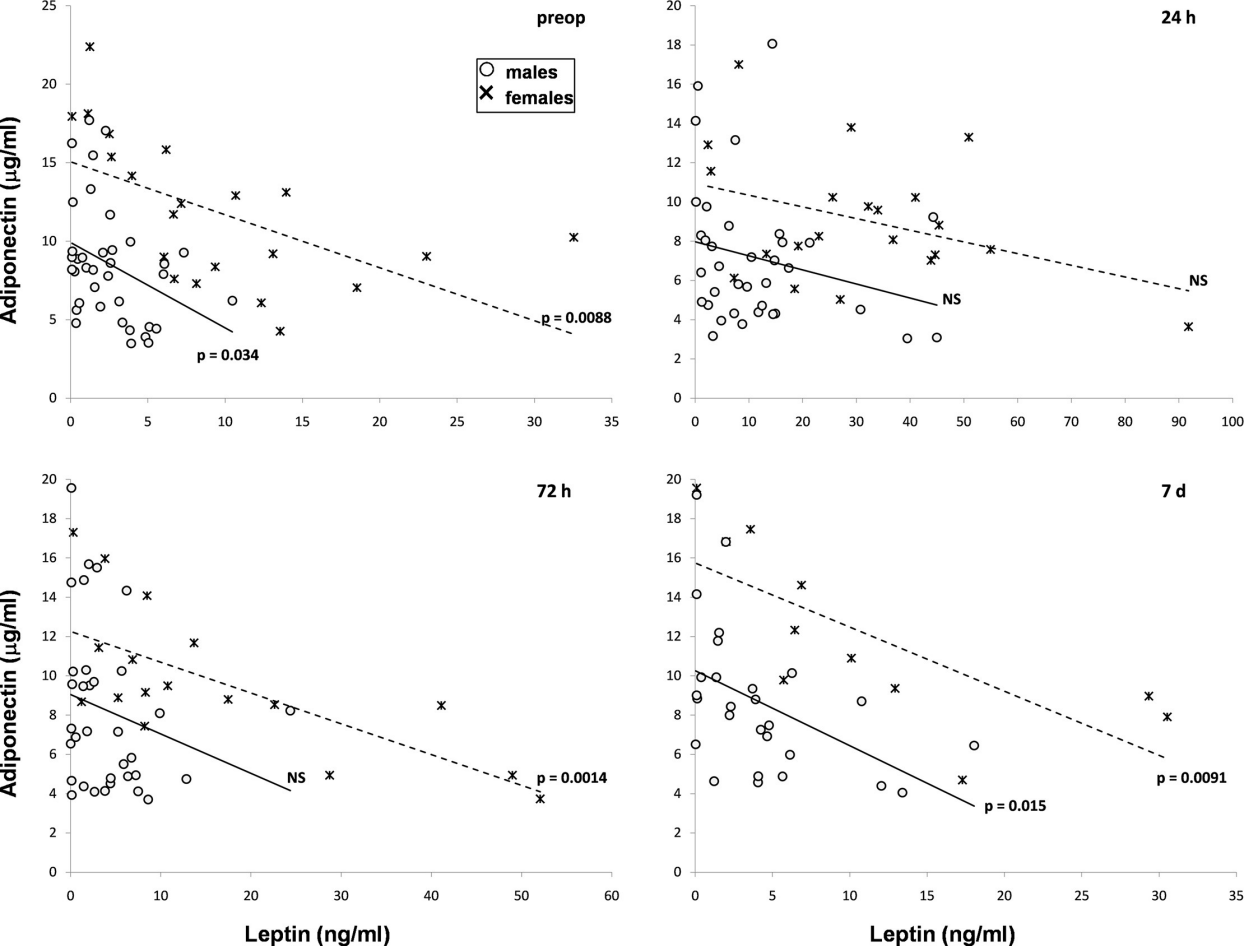
Correlations between serum leptin and adiponectin in males (○) and females (×) with CR cancer. Upper left–before surgery, upper right– 24 hours, lower left– 72 hours and lower right–one week after surgery. Correlation was considered significant at p < 0.05. NS = non-significant.

Leptin and adiponectin levels before surgery were inversely correlated in both male and female RC patients. Correlative significance was lost 24 hours after surgery, but was later regained one week after surgery in males and as soon as 72 hours after surgery in females (Fig 4).

### Hierarchical regression analysis

The correlation of leptin and adiponectin with BW, as well as between leptin and adiponectin were significant before surgery in both males and females, and again at one week after surgery only in males, when neither leptin nor adiponectin were correlated with BW in females. In order to check whether the associations between leptin and adiponectin are independent or confounded by the BW, hierarhical regression analysis was performed (Table 4).

**Table 4:** Results of hierarchical regression.

Gender	Dependent variable	Model	Predictor variable	β	SEE	p
M	adiponectin preop	1	leptin	*-0*.*351*	0.865	*0*.*034*
		2	leptin	-0.265	0.260	0.123
			BW	-0.235	0.055	0.17
	adiponectin 7 d	1	leptin	*-0*.*468*	0.145	*0*.*014*
		2	leptin	-0.378	0.187	0.111
			BW	-0.144	0.071	0.533
F	adiponectin preop	1	leptin	*-0*.*57*	0.112	*0*.*007*
		2	leptin	*-0*.*571*	0.121	*0*.*012*
			BW	0.005	0.053	0.981

M = males, F = females, preop = preoperatory, BW = body weight, β = standardized beta coefficient, SEE = standard error of the estimate, p = significance

Leptin (in the first step) and BW (added to the model in the second step) were entered as predictor variables for adiponectin levels. Standardized β coefficient for leptin decreased and became non-significant when BW was added to the model only in men before and at 7 days after surgery, but not in women before surgery, where the association between adiponectin and leptin remained significant after BW was added in the second step (p = 0.012).

### Influence of tumor localization, nCRT, type of surgery and weight loss on adipokine profile

The rectal tumor was located either in the lower rectum in 30 patients (21 males and 9 females) and in the upper rectum in the other 29 patients (17 males and 12 females). Thirty-two of enrolled RC patients (20 males and 12 females) did not receive nCRT before surgery, whereas 27 patients (18 males and 9 females) received chemoradiation using the long course protocol and operated 60 days ± 10 days after nCRT arrest. Forty-two patients (25 males and 17 females) were operated using LAR type surgery, whereas 17 patients (13 males and 4 females) were submitted to APR. Twenty-five patients (17 males and 8 females) experienced significant weight loss (between 3 and 30 kg in the last year, mean weight loss of 9.5 ± 1.4 kg for all 25 patients, and of 9.9 ± 1.5 and 8.8 ± 3.2 kg for males and females, respectively). The other 34 patients (21 males and 13 females) did not lose weight during the last year before surgery. Leptin and adiponectin had similar dynamics in all subgroups irrespective of tumor localization, the use of nCRT, the surgical technique used or the presence or absence of weight loss. (Table 5).

**Table 5:** Adipokine levels at different tumor localizations, different surgery procedures, and in the presence or absence of presurgical nCRT or weight loss.

Sex	Category	Leptin (ng/ml)				Adiponectin (μg/ml)			
		0	24h	72h	7d	0	24h	72h	7d
M	UR	2.6±0.5	13.5±3.4	4.4±1	5.3±1.7	8.4±0.9	7.2±0.9	8.4±1.2	8.8±2
	LR	3.1±0.6	11±2.6	4.9±1.4	4.3±1.1	8.7±1	7.1±0.8	8.2±1	8.5±0.9
	p	0.314	0.784	0.591	0.516	0.631	0.957	0.312	0.404
	LAR	2.5±0.4	13.2±2.5	4.2±0.8	5±1.2	8.8±0.8	7.3±0.8	8.5±1	8.8±1
	APR	2.8±0.9	8.4±3.2	4±1.8	2.7±0.8	8±0.9	6.9±0.9	8.2±0.9	8.2±0.9
	p	0.764	0.148	0.958	0.405	0.643	0.684	0.441	0.312
	nCRT-	2.1±0.4	8.8±2.3	2.8±0.6	3.5±1.1	9.4±1	7.9±0.4	8.1±1	8.1±0.9
	nCRT+	3.2±0.7	14.9±3.2	5.8±1.7	5.1±1.5	7.5±0.5	6.2±0.5	8.4±1.1	9.3±1.1
	p	0.099	0.215	0.137	0.689	0.136	0.218	0.679	0.081
	W-	2.2±2.4	12.1±2.9	3.5±1	4.7±2	8.7±1	7.4±1	8.5±1.3	9±1.4
	W =	2.3±0.6	11.1±2.8	4.6±1.2	4±0.9	8.3±0.8	7±0.7	8±0.9	8.5±0.9
	p	0.55	0.995	0.523	0.71	0.742	0.946	0.818	0.551
F	UR	10.6±3.2	27±5.5	11.6±2.9	12.3±4.1	12.6±1.7	9.8±1.2	10.8±1.1	11.6±1.9
	LR	9±2.4	26.4±5.8	27.5±11.3	13.7±8.5	11.4±1.4	8.9±0.9	8.4±2.1	13.3±2.8
	p	0.696	0.943	0.074	0.871	0.603	0.557	0.311	0.639
	LAR	9.5±1.9	32.3±5.3	15.5±4.2	10.1±3	12.2±1.1	9.3±0.8	9.9±1	11.9±1.5
	APR	9.8±4.7	25.5±9.2	24.7±16.4*	17.1±13.5*	10.5±2.6	8.1±1.3	8±0.5*	12.7±4.8*
	p	0.739	0.284	0.743*	0.947*	0.833	0.555	0.279*	0.902*
	nCRT-	10.9±2.8	30±7.1	17.9±6	10.4±3.5	11.9±1.5	9±1	9.8±1.4	12.4±1.8
	nCRT+	7.3±1.5	30.1±5.6	9.8±2.5	14±7.7	11.6±1.5	8.8±0.9	10.4±0.9	11.1±1.8
	p	0.151	0.909	0.436	0.777	0.944	0.793	0.425	0.982
	W-	7.7±3.9	21.7±6	12.5±6.2	9±7.2	14.6±1.8	10.9±1.2	11.7±1.6	15.8±1.3
	W =	10.6±1.6	36.8±6	18.8±5.3	12.7±3.2	10.2±1	8±0.7	8.6±1	9.7±1.1
	p	0.336	0.101	0.123	0.306	***0*.*028***	***0*.*043***	***0*.*004***	***0*.*021***

M-males, F-females, UR–tumor of the upper rectum, LR–tumor of the lower rectum, LAR–patients operated by low anterior resection, APR–patients operated by abdomino-perineal resection. nCRT- patients not receiving chemotherapy, nCRT+ patients receiving chemotherapy. W- patients experiencing weight loss, W = patients with no weight loss.

* not interpretable due to low number of patients left in the group (only 2). No significance was found at any of the time points excepting women having weight loss, who had significantly higher adiponectin (p<0.05, bold italic).

**Fig 5:**
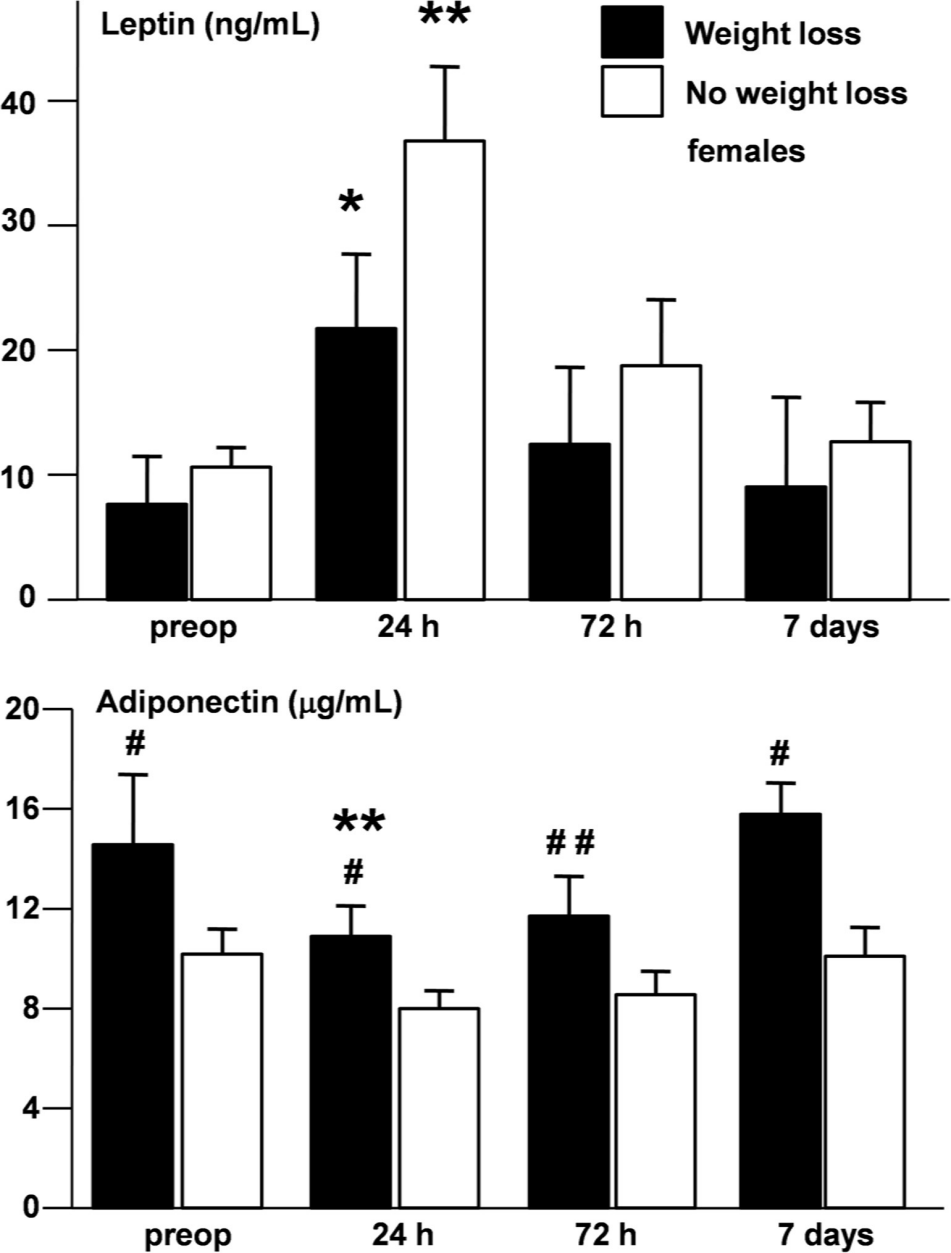
Leptin and adiponectin levels in women with weight loss. Upper panel–leptin, lower panel–adiponectin, white bars–women without weight loss, black bars–women with weight loss, * compared to presurgical levels, # compared to women without weight loss, * or # p < 0.05, ** or ## p < 0.01.

There were no significant differences between adipokine levels at different time points in both sexes, except for adiponectin, which had significantly higher levels at women who lost weight (Fig 5).

## Discussion

The onset and evolution of CRC are influenced by pleiotropic factors: genetic background [26], but also many other variables, such as diet [27], smoking [19] or even fine tuning environment factors such as selenium or other antioxidants [28,29]. The adipocyte is a resource of hormones involved in inflammation [1]. Both obesity and an inflammatory environment are correlated with the appearance of various cancers [30], including CRC [2–4]. The majority of our RC patients were indeed overweight or obese (Table 1).

CRC cells possess receptors for leptin and adiponectin [11,31,32]. Leptin was shown to have oncogenic effects [2,31,33,34], whereas adiponectin demonstrated opposite, anti-inflammatory and differentiating actions [2,32,34]. These adipokines were therefore proposed among vectors linking adiposity and CRC [2–4]. Trying to use serum levels of leptin and adiponectin as prognostic markers in CRC lead, however, to conflicting results. Since leptin proved oncogenic and adiponectin anti-oncogenic effects, one would expect that CRC should be associated with high leptin and low adiponectin levels. Such an adipokinic profile in patients with CRC was indeed described by several authors [2–4,6–8,15,16]. Other studies obtained, however, opposite conclusions, with low leptin levels in patients with CRC [9–13], whereas certain investigators did not observe significant CRC-related changes of leptin [4,14] or adiponectin [17,18]. This discordance may be caused by different localizations of CRC in study-enrolled patients [18,19,21,22], by cancer staging [8,11,14] and multicenter approach [19–23].

Taking into consideration the high variability of literature data, we wanted to check the modifications of leptin and adiponectin secretion exclusively in rectal cancer, which is often diagnosed in pre-metastatic stages and has a higher survival rate [35]. We enrolled all our study participants from the same surgical center and compared their leptin and adiponectin levels with weight and age matched healthy controls.

Women had higher leptin and adiponectin serum concentrations in both RC and healthy group pairs, as described by others [36]. It was therefore important to evaluate adipokine modifications distinctly in men and women, comparing groups of the same gender.

Our RC patients had lower oncogenic leptin and higher anti-oncogenic adiponectin than gender, weight and age matched controls. These results suggest that changes of the adipokinic profile of RC patients may correspond to an adaptation to the malignant condition rather than an obesity-related triggering factor for malignancy. Lower leptin in CRC was also observed by certain investigators [9–13], in contrast to others [3,6–8]. Literature data portray a beneficial, protective role of adiponectin against digestive malignancy [3–5]. Contrary to us, a subgroup analysis of the EPIC study on 186 RC patients described lower adiponectin levels than their corresponding controls [3]. Data was however pooled from both men and women diagnosed with RC. Moreover, the authors did not provide information with respect to cancer staging heterogeneity, whereas all our RC volunteers had similar disease staging (stage 2 and 3 RC).

We further wanted to investigate the secreting profile of the two adipokines after surgery and observed a significant leptin increase and adiponectin decrease 24 hours after surgery in both men and women, followed by a recovery to initial values at 72 hours and 7 days after surgery. It is known that surgical stress greatly influences adipokine profile as the result of an inflammatory adaptive process [37]. Other investigators observed similar adipokine modifications not only after surgery for cancer [38–40], but also in other types of stress caused by surgery or critical state [41]. These changes are therefore related more with stress by itself and less with the stress triggering factor. The amplitude of these changes until return to initial level has, however, individual variation and may reflect adaptive particularities with prognostic potential (e.g. blunted postoperatory leptin increase meaning the overlap of sepsis and/or a higher mortality risk) [42].

In order to evaluate the prognostic potential of adipokine measurement, we further investigated the influence of tumor localization, presurgical nCRT, surgical technique or weight variation on postsurgical adipokine dynamics and observed significantly higher levels of adiponectin only in women with RC who experienced significant weight loss during the last year before surgery.

Other investigators demonstrated a significant impact of chemotherapy on adipokine levels in CRC [24]. Their patients had, however, advanced forms of heterogeneously localized CRC and were submitted to chronic, 5 alpha fluorouracil-based palliative chemotherapy. All our patients had only RC and were operated, some being submitted only to a limited period of nCRT before surgery, which did not have a significant impact on adipokine profile. Surgical techniques vary function of tumor localization in RC, with LAR type surgery being accompanied by a less traumatizing and stressful recovery than APR [43]. This well described difference was, however, not reflected by postsurgical adipokine variability, which was similar in patients submitted to LAR or APR. Adipokine profile seems therefore unsuited to discriminate postsurgical evolution of RC patients.

Women who experienced weight loss during one year prior surgery had significantly higher adiponectin levels before surgery and at all time points after surgery. Other authors described leptin and adiponectin modifications with variations in BW [44–46]. We did not observe different leptin levels in women or men who lost weight. Contrary to women, men who lost weight did not have different adiponectin levels. These data plead in favor of gender-determined differences in secreting behavior of adipose tissue with weight loss, as observed by others [45,46]. It is, therefore, unclear whether this gender difference is related to RC or is common to weight variations irrespective of their causes.

Preoperatory leptin and adiponectin were directly and respectively inversely correlated with BW in both males and females with RC. Fat tissue represents an important body compartment, tightly related to BW. Leptin is directly secreted by adipose cells and reflects therefore total fat mass in the conditions of stable energy balance [42]. Although also secreted by white adipose cells, adiponectin is inversely correlated to fat mass. The inhibition of adiponectin expression is triggered by higher energy intake rather than by the adiposity per se [47].

Various types of stress or inflammatory processes interact with adipokine secretion irrespective of fat mass [48]. We wanted to evaluate therefore the maintenance or distortion of adipokine correlation with body mass by the stress caused by surgical intervention. Surprisingly, whereas the correlation between adipokines and BW was conserved significant in all time points in males, it became non-significant in females not only 24 hours after surgery, but also at later time points, up to one week after surgery. The reaction of adipose tissue to surgical stress in RC patients seems therefore to display a gender divergence, possibly related to differences in hormonal milieu [49] or fat tissue distribution [36]. These observed gender differences in adipokine correlations with BW may have also been influenced by the smaller size of the female subgroup.

Interestingly, we observed an inverse correlation between leptin and adiponectin before surgery, which became non-significant 24 hours after surgery in both males and females. Significance was restored at 72 hours in females and only after 7 days in males. Since volume (mass) of adipose tissue is directly correlated with leptin and inversely correlated with adiponectin [50], it is not unexpected to observe an inverse correlation also between leptin and adiponectin. The disturbance of this correlation 24 hours after surgery may be related to the interference of a supplementary factor with adipokine secretion, which is probably stress [37–41].

Leptin, adiponectin and BW were significantly interrelated before surgery in both genders. The correlation between BW and adipokines was preserved in males at 24, 72 hours and 7 days after surgery, but became non-significant in females. The three parameters were again all associated significantly in simple correlations at 7 days after surgery only in males. We wanted to observe the interaction of leptin, adiponectin and BW in patients with RC by using hierarchical regression analysis in order to identify the role of BW as a possible confounder between the two adipocytokines according to gender differences.

Hierarchical regression analysis showed the disappearance of significance in the second step (when BW was added) before surgery and after 7 days only in males. In presurgical samples, females preserve the significance of the adiponectin—leptin association independently of BW (the second step of hierarchical regression). These data point out that the association of leptin with BW is a confounding factor for adiponectin in males, but that adiponectin secretion is influenced by leptin independently of BW in females. Interestingly, although adiponectin and leptin keep being correlated significantly at 72 hours and one week after surgery in females, none of the two adipokines maintain a significant association with BW. These results suggest again that adiponectin and leptin may have a significant interference in women, which is independent of BW. Literature data demonstrate antagonistic effects of leptin and adiponectin [2,4,51,52], but fail to describe a direct interference of any of these adipokines with the secretion of the other [53]. Leptin and adiponectin may, however, influence each-other’s levels through opposite modifications in insulin sensitivity [49,53,54], although the precise sequence of events is not clear. Fasting glucose, insulin and HbA1c were, however, not assessed in our study.

Our study has several strengths, but also limitations. Study originality consisted in that all our enrolled patients had a type of digestive cancer localized in the same region–the rectum. The patients were also recruited from the same clinical center and were all operated by the same surgical team, clearly making this study more homogenous than others [3,4,6–9,11,12,18,21,22]. Study drawbacks included the limited number of volunteers, with fewer females with RC and short follow up period due to high dropout rate. This limitation made the evaluation of adipokines as prognostic markers hard to be performed. Evaluated cytokinic spectrum was moreover limited to leptin and adiponectin, other adipokines or fat tissue-related markers (soluble leptin receptor, adiponectin isoforms, resistin, etc.) not being assessed. Finally, fat tissue mass or its distribution was again not assessed in our volunteers.

## Conclusions

We observed a particular adipokinic spectrum in RC patients compared to healthy age-, BW- and sex-matched controls, with lower leptin and higher adiponectin, possibly as an adaptation of adipose tissue to this critical situation (Table 2). Surgical stress produced important modifications of adipokine secretion, which came back to initial values within one week (Fig 2).

Adipokine secretion behaved differently in the two genders suffering of RC. Both leptin and adiponectin levels were higher in women than in men (Fig 2). The correlation between adipokines and BW persisted in men but became non-significant in women up to one week after surgery (Table 3 and Fig 3). Hierarchical regression showed the presence of a significant association between leptin and adiponectin only in females which was independent of BW (Table 4 and Fig 4). The fat mass-independent relationship between the two adipokines in women may be mediated by their particular interference with other metabolic loops. Finally, adiponectin was significantly higher in women, but not men with weight loss, when compared to patients without weight loss of the respective gender (Table 5 and Fig 5). Energy metabolism may therefore interfere in another way with adiponectin secretion in females, possibly due to their hormonal background, but also to gender differences in body composition and fat tissue distribution.

These data strengthen the need for further research in evaluating the potential of adipokines as prediction factors in various disease states.

## Supporting information

S1 File Raw data healthy vs RC patients. Age, sex, weight, leptin, adiponectin in healthy volunteers and RC patients.
(XLSX)

S2 File Raw data RC patients. Weight variation in the last year, chemo/radiotherapy, tumor localization, type of surgery, leptin and adiponectin levels after surgery in RC patients.
(XLSX)
